# Acute thoracic disc herniation with severe spinal cord compression: a case report

**DOI:** 10.1093/jscr/rjaf001

**Published:** 2025-01-15

**Authors:** Mario Cahueque, Andres Cobar, Santiago Montenegro

**Affiliations:** Hospital Maranatha, 13 Calle 15, Guatemala City 01010, Guatemala; Hospital Centro Medico, 6A Avenida 3-47, Guatemala City 01010, Guatemala; Universidad Francisco Marroquín, 6A Calle final, Guatemala City 01010, Guatemala

**Keywords:** acute thoracic disc herniation, herniated disc, transpedicular approach, microdiscectomy, spinal decompression

## Abstract

This case report highlights the rare presentation of an acute thoracic disc herniation in a 27-year-old male. Thoracic disc herniations are uncommon, accounting for less than 1% of all disc herniations, and acute presentations have scantly been recorded in literature. The patient, a mechanic, presented with a sudden onset of dorsal pain and bilateral lower limb weakness after lifting heavy equipment, leading to a sudden cease of most motor functions in the patient’s lower limbs. Magnetic resonance imaging revealed a severe T9/T10 herniation with significant spinal cord compression. Emergency surgical decompression via a right-sided transpedicular thoracic approach was performed, resulting in progressive neurological recovery. This case underscores the importance of early diagnosis, timely surgical intervention, and multidisciplinary management in achieving favorable outcomes for this rare condition.

## Introduction

Thoracic disc herniations (TDH) are a rare entity in spinal pathologies, representing <1% of all herniated discs [1, 2]. Unlike cervical or lumbar disc herniations, TDH is often asymptomatic or presents with vague symptoms, making diagnosis challenging; the prevalence of symptomatic thoracic herniations is as low as 1 in 1 000 000 patients per year [3]. Acute TDH with spinal cord compression, as in this case, is exceptionally rare, with only a limited number of cases reported in the literature [4]. Chronic TDHs, often calcified, are more commonly described, but acute cases with sudden neurological deficits demand urgent attention. This report discusses the clinical presentation, diagnostic approach, surgical management, and postoperative course of an acute T9/T10 TDH in a young patient following a certain degree of trauma to the spine. The case highlights the importance of early intervention and multidisciplinary rehabilitation to maximize recovery in rare and potentially debilitating conditions.

## Case presentation

**Figure 1:**
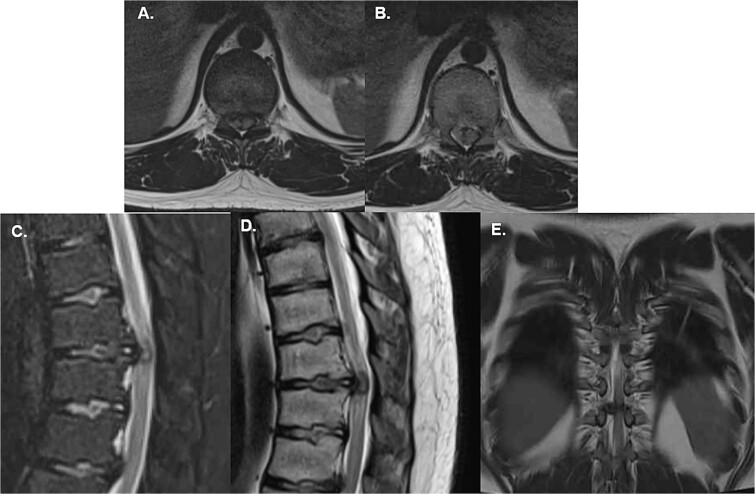
Emergency MRI of the patient shows (A) and (B) axial T2W projections that demonstrate a severe compression of the spinal cord given the central location of the extruded disc, (C) a sagittal T2W with fat suppression allows to see the extruded disc nucleus of the T10-T11 disc causing compression of the spinal cord; edematous changes are also visible, (D) shows a T1W sagittal midline image of the extruded disc which also shows a flattened spinal cord with associated edema; note the absence of any calcification or changes associated with chronic progression and (E) is a coronal T2W image of the disc herniation showing its central location with a slight predominance over the right canal.

A 27-year-old male mechanic with no significant medical history presented to the emergency department after an acute event of dorsal pain and bilateral lower limb weakness. The patient reported lifting a heavy piece of equipment, when he experienced a sudden, sharp pain in the mid-back, followed immediately by a loss of strength in both legs. The weakness caused him to collapse to the ground, and he was unable to stand or walk thereafter. Upon arrival at the emergency department, the patient was alert and hemodynamically stable but exhibited profound motor deficits in the lower extremities. The relevant findings to the physical exam included motor function assessment showing 1/5 strength bilaterally in the lower limbs with sensory function intact across all dermatomes (Supplementary Video 1). The patient was unable to void spontaneously, and a Foley catheter was placed to relieve urinary retention. However, he did present with a certain degree of sacral preserving, accounting for spontaneous erections and preserved defecation. An urgent MRI of the thoracic spine revealed a large, extruded disc herniation at T9/T10, causing severe compression of the spinal canal. The spinal cord appears flattened at the level of herniation, with significant associated edema. No evidence of calcification or chronic changes was noted, suggesting an acute pathology (Fig. 1).

### Management

**Figure 2:**
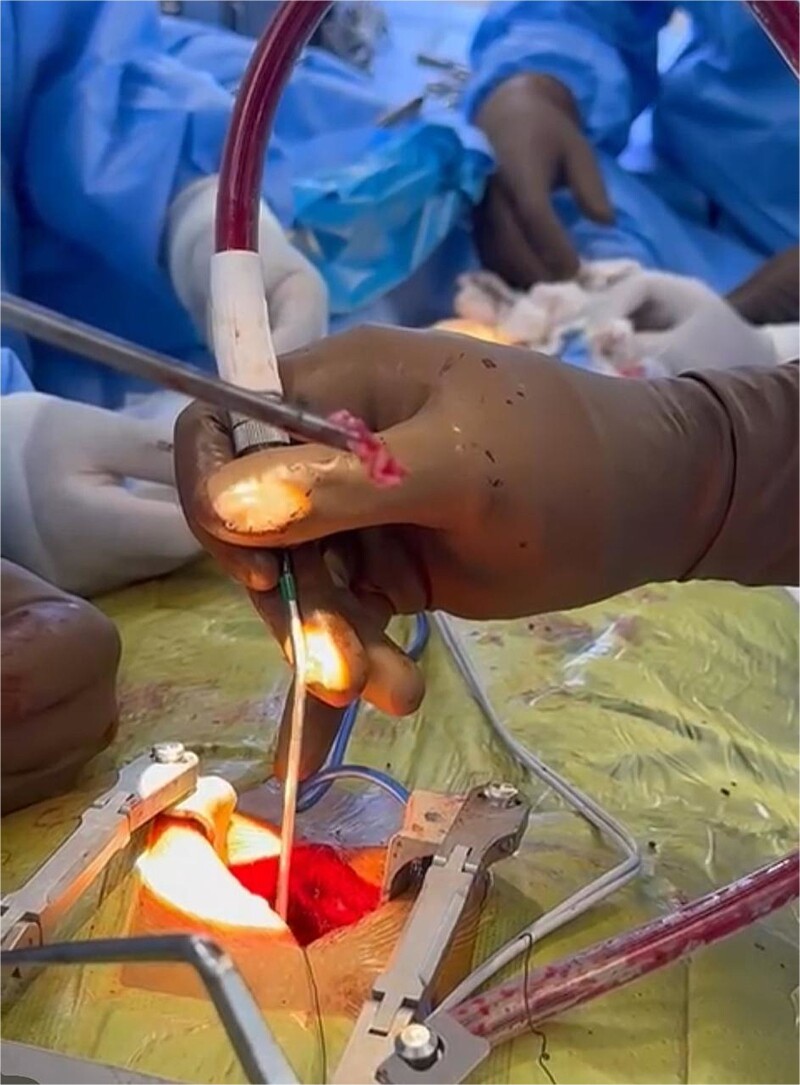
Intraoperative image of the herniated material removed from the spinal canal.

Given the acute onset of symptoms and severe neurological compromise, the patient was taken for emergency surgical decompression. The surgical team performed a microdiscectomy and spinal cord decompression using a right-sided transpedicular thoracic approach. This technique was chosen to provide direct access to the herniated disc while minimizing disruption to surrounding structures [5, 6]. Intraoperative findings included a large, soft disc fragment that was identified and removed, resulting in immediate decompression of the spinal cord (Fig. 2). The procedure duration was approximately 3 hours, and no intraoperative complications were reported. Postoperatively, the patient was transferred to the recovery unit in stable condition.

### Postoperative course

The patient's recovery was closely monitored, with serial neurological assessments performed to evaluate motor and sensory function. During the first 48 hours of postoperative recovery, the patient remained stable, and no additional neurological deficits were noted. Pain was managed effectively with analgesics, and he was mobilized in bed under the supervision of the physical therapy team. The patient was discharged home 48 hours postoperatively with a Foley catheter in place for bladder management. Two weeks postoperative the patient had his first office visit; the Foley catheter was successfully removed after the patient regained bladder control. Neurological examination revealed initial motor recovery, with strength in the lower limbs improving to 2/5. Sensory function remained intact, and sacral sparing was preserved. The patient began outpatient physiotherapy, focusing on strengthening and mobility exercises. By the sixth week, the patient demonstrated significant improvement. Muscle strength in the lower limbs improved to 3/5, allowing for movement of the lower limbs against gravity. With the aid of a cane, the patient was able to walk short distances (Supplementary Video 2). Video documentation was taken during this visit, showcasing his progress. The patient continues to attend regular physiotherapy sessions, showing gradual improvement in motor strength and coordination, on a course to full recovery and independence.

## Discussion

TDHs are a rare clinical entity, with acute presentations being even more uncommon. Chronic calcified herniations are the most frequently reported in the literature, often associated with gradual onset of symptoms such as thoracic radiculopathy, myelopathy, or both [2, 7]. In contrast, acute herniations, as seen in this case, can present dramatically with sudden neurological compromise requiring urgent intervention. TDHs occur most commonly in the lower thoracic spine, between T8 and T12, due to increased biomechanical stress in this region. The relative rarity of symptomatic TDH is attributed to the stability provided by the rib cage, the narrow thoracic spinal canal, and the relatively small intervertebral space and discs, which allows less space for herniated material to compress the spinal cord [1, 8]. Surgical decompression is the cornerstone of treatment for acute TDH with significant neurological deficits. The transpedicular approach used in this case allowed direct access to the herniation while preserving the stability of the thoracic spine; historically, this approach has been strongly recommended to achieve access to the posterior aspect of the vertebral body [9]. The prompt intervention likely contributed to the patient’s favorable recovery trajectory [5, 10]. Neurological recovery following acute TDH depends on the timing of intervention and the degree of preoperative neurological compromise. This case highlights the potential for significant improvement, even in severe cases, provided that decompression is performed promptly and is followed by a structured rehabilitation program.

## Conclusion

This case underscores the rarity and clinical significance of acute TDH with spinal cord compression. While chronic thoracic herniations are more commonly described, acute presentations pose unique diagnostic and therapeutic challenges. Early recognition, timely surgical intervention, and comprehensive rehabilitation are essential to achieving favorable outcomes.

This report contributes to the limited body of literature on acute TDH, serving as a valuable reference for clinicians managing similar cases.

## Supplementary Material

Supplementary_Video_1_rjaf001

Supplementary_Video_2_rjaf001
